# Supplementary material for: Sirtuin 2 Dysregulates Autophagy in High-Fat-Exposed Immune-Tolerant Macrophages
Source: Cells. 2021 Mar 26;10(4):731. doi: 10.3390/cells10040731 (PMC8066127; doi:10.3390/cells10040731)
Supplement: Supplementary file 1 [file cells-10-00731-s001.pdf]

cells

MDPI

Article

Sirtuin 2 Dysregulates Autophagy in High-Fat Exposed Immune Tolerant Macrophages

Sanjoy Roychowdhury [1,†], Anugraha Gandhirajan [1,†], Christopher Kibler [1], Xianfeng Wang [2] and Vidula Vachharajani [1,3,*]

Supplmentary figures:

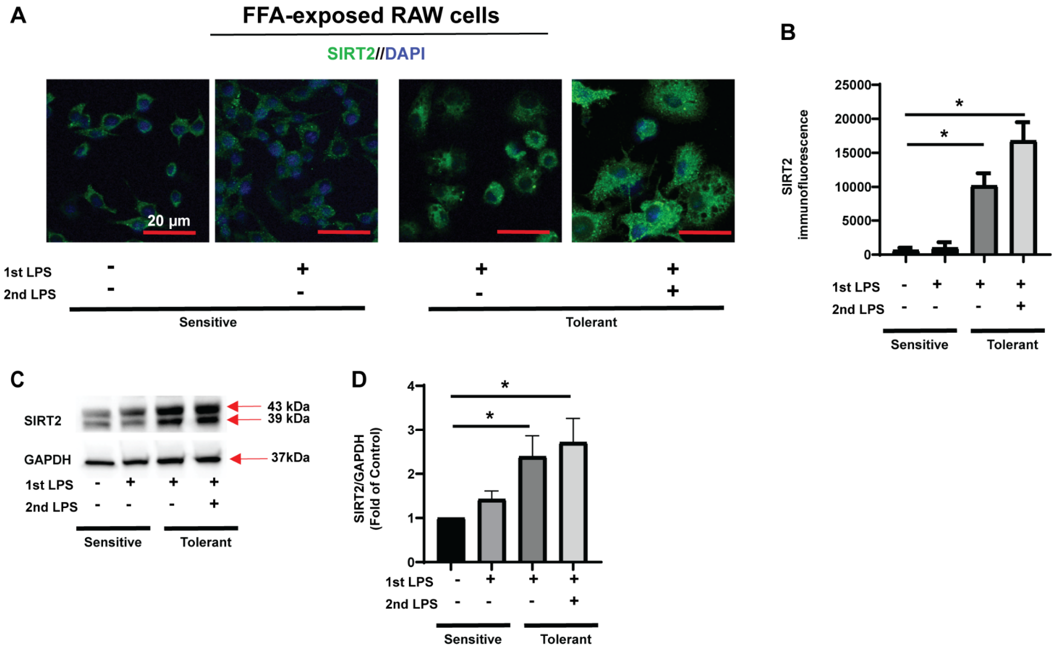

Figure S1. SIRT2 expression is increased in FFA-exposed tolerant RAW cells. Stearic acid (free fatty acid: FFA) exposed RAW264.7 cell macrophages (RAW) were stimulated with either one or two doses of LPS. A. Representative images of SIRT2 (green) and DAPI (blue) immunostaining in FFA-exposed sensitive and tolerant RAW cells following one or two doses of LPS stimulation as indicated. **B.** Fluorescence quantification of SIRT2 immunostaining in FFA-exposed sensitive and tolerant RAW cells (n = 5; * $p < 0.05$). **C.** SIRT2 protein expression was detected by Western Blot in FFA-exposed RAW cells following one or two doses of LPS stimulation as indicated. GAPDH was used as the loading control. **D.** Western blot image quantification using image-J software (n = 3 each group; * $p < 0.05$). SIRT2 protein expression was normalized to GAPDH.

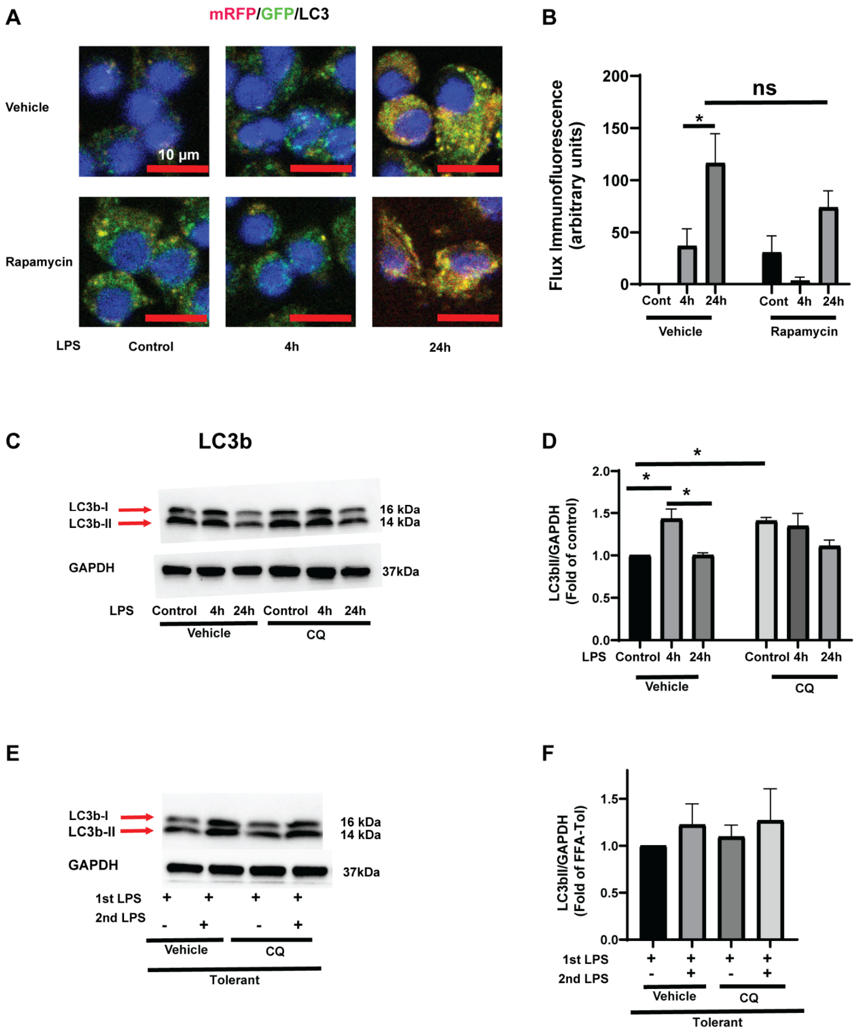

Figure S2. Autophagosome accumulates in FFA-exposed tolerant RAW cells Stearic acid (free fatty acid: FFA) exposed RAW264.7 cell macrophages (RAW) were stimulated with either one or two doses of LPS as indicated time points. **A**. To induce autophagic clearance, cells were treated with rapamycin (100 ng/ml, 24 h). Representative images of autophagosome formation (yellow puncta) with mRFP-GFP-LC3 transduction in FFA-exposed sensitive RAW cells. **B**. Fluorescence quantification of mRFP/GFP fluorescence in FFA-exposed RAW cells in control (without LPS) and LPS. **C**. To inhibit autophagy, cells were exposed to chloroquine (90 µM, 24 h). Loading control: GAPDH. LC3b protein expression was detected in the cytosolic extract by Western Blot. **D.** Western blot image quantification of LC3b-II using image-J software (n = 5 blots; * $p < 0.05$). **E**. FFA exposed tolerant RAW cells were treated with autophagy inhibitor CQ (90 µM) or vehicle and incubated for 24h and stimulated with or without LPS as indicated. LC3b protein expression was detected in the cytosolic extract by Western Blot. **F.** Western blot image quantification of total alpha-tubulin using image-J software (n = 5 blots; * $p < 0.05$).

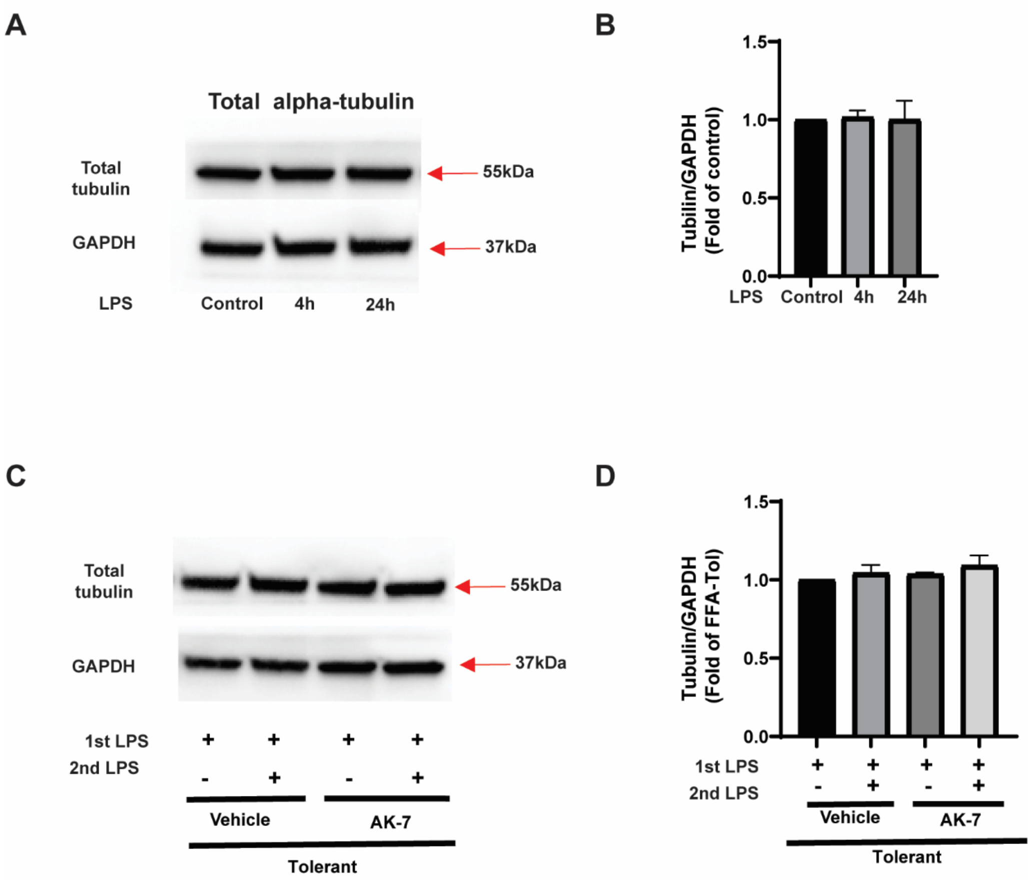

Figure S3. Total alpha-tubulin expression in sensitive and tolerant macrophages. Stearic acid (free fatty acid: FFA) exposed sensitive and tolerant RAW264.7 cell macrophages (RAW) were stimulated with or without LPS for 4h or 24 h. Loading control: GAPDH. **A.** Total alpha-tubulin protein expression was detected in the cytosolic extract by Western Blot. **B.** Western blot image quantification using image-J software (n = 5 blots; * $p < 0.05$). **C** FFA exposed tolerant RAW cells were treated with SIRT2 inhibitor AK-7 (25 μM) or vehicle (DMSO; equal volume) and incubated further for 20h and stimulated with or without LPS as indicated. Total alpha-tubulin protein expression was detected in the cytosolic extract by Western Blot. **D.** Western blot image quantification of total alpha-tubulin using image-J software (n = 5 blots; * $p < 0.05$).

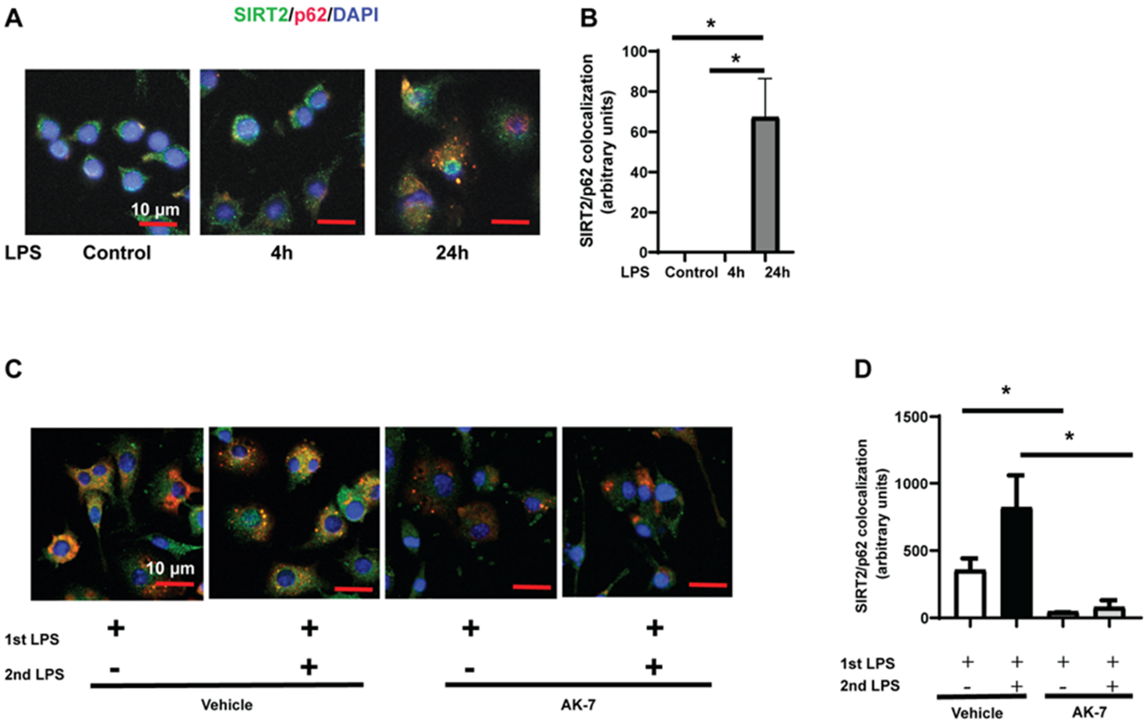

Figure S4. Co-localization of SIRT2 and p62 in sensitive and tolerant macrophages. Stearic acid (free fatty acid: FFA) exposed sensitive and tolerant RAW264.7 cell macrophages (RAW) were stimulated with or without LPS as indicated. **A.** Representative Images of SIRT2 (green) and p62 (red) double immunostaining in FFA-exposed sensitive and tolerant RAW cells following LPS stimulation as indicated. Yellow color indicates co-localization of SIRT2 and p62. **B.** Fluorescence quantification of SIRT2 and p62 double immunostaining in FFA-exposed sensitive and tolerant RAW cells. **C.** FFA exposed tolerant RAW cells were treated with SIRT2 inhibitor AK-7 (25 μM) or vehicle (DMSO; equal volume) and incubated further for 20h and stimulated with or without LPS as indicated. Representative Images of SIRT2 (green) and p62 (red) double immunostaining in FFA-exposed sensitive and tolerant RAW cells following LPS stimulation as indicated. **D.** Fluorescence quantification of SIRT2 and p62 double immunostaining in FFA-exposed tolerant RAW cells in presence or absence of AK-7.
